# Experimental Study on the Strength Deterioration and Mechanism of Stabilized River Silt Reinforced with Cement and Alginate Fibers

**DOI:** 10.3390/ma17133124

**Published:** 2024-06-26

**Authors:** Ying Wang, Chaojie Wang, Zhenhua Hu, Rong Sun

**Affiliations:** 1 College of Transportation, Shandong University of Science and Technology, 579 Qianwan Port Road, Qingdao 266590, China; wangying871209@sdust.edu.cn (Y.W.); 202183160020@sdust.edu.cn (C.W.); 2 College of Civil Engineering and Architecture, Shandong University of Science and Technology, 579 Qianwan Port Road, Qingdao 266590, China; 202383040048@sdust.edu.cn

**Keywords:** stabilized soil, cement, alginate fiber, river silt, strength deterioration, reinforcement mechanism, deterioration mechanism

## Abstract

River silt deposited by water in coastal areas is unsuitable for engineering construction. Thus, the in situ stabilization treatment of river silt as the bearing layer has been an important research area in geotechnical engineering. The strength degradation behavior and mechanism of stabilized river silt reinforced with cement and alginate fibers (AFCS) in different engineering environments are crucial for engineering applications. Therefore, freeze–thaw (F–T) cycle tests, wetting-drying (W–D) cycle tests, water immersion tests and seawater erosion tests were conducted to explore the strength attenuation of stabilized river silt reinforced with the same cement content (9% by wet weight) and different fiber contents (0%, 0.3%, 0.6% and 0.9% by weight of wet soil) and fiber lengths (3 mm, 6 mm and 9 mm). The reinforcement and damage mechanism of AFCS was analyzed by scanning electron microscopy (SEM) imaging. The results indicate that the strength of AFCS was improved from 84% to 180% at 15 F–T cycle tests, and the strength of AFCS was improved by 26% and 40% at 30 W–D cycles, which showed better stability and excellent characteristics owing to the hygroscopic characteristics of alginate fiber arousing the release of calcium and magnesium ions within the alginate. Also, the strength attenuation of AFCS was reduced with the increase in the length and content of alginate fibers. Further, the strength of specimens in the freshwater environment was higher than that in the seawater environment at the same fiber content, and the softening coefficient of AFCS in the freshwater environment was above 0.85, indicating that the AFCS had good water stability. The optimal fiber content was found to be 0.6% based on the unconfined compressive strength (UCS) reduction in specimens cured in seawater and a freshwater environment. And the strength of AFCS was improved by about 10% compared with that of cement-stabilized soil (CS) in a seawater environment. A stable spatial network structure inside the soil was formed, in which the reinforcing effect of fibers was affected by mechanical connection, friction and interfacial bonding. However, noticeable cracks developed in the immersed and F–T specimens. These microscopic characteristics contributed to decreased mechanical properties for AFCS. The results of this research provide a reference for the engineering application of AFCS.

## 1. Introduction

River silt consists of fine-grained sediments with a particle size between that of sand and clay, typically between 0.002 mm and 0.06 mm in diameter. River silt particles are generally larger than clay particles but smaller than sand particles, and they are often found deposited by water in areas such as riverbeds or floodplains. River silt can significantly impact the environment and human activities, such as by affecting water quality, reducing soil fertility and causing problems for navigation, irrigation and construction. Thus, their engineering application is challenging due to the undesirable characteristics of a high moisture content, a lower compression modulus, high rheology, low strength and low permeability [1,2,3,4]. Most river silt is treated with landfill, dumping, sea dumping and incineration, which not only occupy a large quantity of land resources but also cause pollution in groundwater and soil resources. According to the Global Environment Facility (GEP) Research report, the global annual output of river silt exceeds 100 million tons, of which the annual output in the United States, European Union and China has exceeded 35 million tons, 40 million tons and 50 million tons, respectively. The annual output of river silt in the Taihu Basin in China alone exceeded 7 million tons [1]. With increasing river silt production, the rational utilization of river silt, turning waste into treasure, has gradually attracted attention among researchers.

River silt stabilization technology is mainly categorized into physical dehydration consolidation, high-temperature dissolution and bonding, and chemical stabilization treatment [5,6,7,8,9,10,11,12,13]. The first two methods are primarily used on occasions with small dosages and high requirements. In chemical stabilization treatment, treated river silt is converted into construction materials by adding some stabilized materials through specific physical and chemical methods [12,13], which can not only effectively solve the problem of river silt but also alleviate the issue of land resource shortages. In addition, it is suitable for large-scale engineering applications. In the face of a large amount of river silt waste, stabilizing river silt treatment technology has an excellent prospect from the perspective of technical requirements and application range. Based on the current research, stabilized river silt can better solve the problems of significant moisture content, high compressibility and low strength, thus reducing deformation, improving strength and stability and meeting engineering performance requirements [2,3,14,15,16,17,18]. Stabilized river silt in situ can quickly form a bearing platform, providing a site for subsequent construction, and the construction period can be significantly reduced compared with the drainage method.

Some problems are faced by solely using cement-stabilized agents, such as tension cracks, disintegration or collapse caused by soaking in water, fracturing and cracking caused by moisture loss, and brittle fracture or failure. Hence, meeting the requirements for complex engineering applications and engineering stability is challenging. Thus, some researchers have synergistically used fibers and inorganic stabilized agents to treat river silt. Zhang et al. [19,20], Munoz et al. [21], Sahlabadi et al. [22] and Lu et al. [23] found that the mechanical properties of cement-stabilized soil incorporating fibers were improved compared with using cement alone. Himouri et al. [24] showed that the anti-drying shrinkage, mechanical strength and water absorption performance of stabilized soil improved with Date Palm fibers and cement were better than those of solely using cement. The above study found that the mechanical properties of cement-stabilized soil incorporated with fibers were enhanced [19,20,21,22,23,24]. In addition, in some research, the strength deterioration of cement-stabilized soil reinforced with fibers was reduced, and the durability was enhanced [5,25,26,27,28,29]. Roshan et al. [25,26] studied the durability of fiber-reinforced cement-stabilized soil through different tests by adding polypropylene fibers into stabilized sand. Tiwari et al. [5,27] explored its coupled effect on controlling the strength and durability of expansive soil by using different fibers to improve stabilized soil. Akbari et al. [28] studied its mechanism of action through drying–wetting cycle tests and SEM imaging by improving lime-stabilized soil with nano-zeolite and polypropylene fibers. Kumar et al. [29] explored the influence of different sugarcane bagasse fiber contents on the mechanical strength and durability of stabilized soil.

From the development point of sustainability, the application of bio-based fibers for soil stabilization has advantages. Compared with other natural fibers, alginate fiber with softness, moisture absorption and air permeability is extracted from ocean algae, having better applicability for the environment and belonging to the polysaccharide class of substances. As a new type of biological fiber, alginate fiber has certain functions compared with other natural fibers [30,31] in terms of metal ion adsorption, flame retardancy, high moisture absorption, far infrared and anion function, and high oxygen permeability. Especially, due to the hygroscopic characteristics of alginate fiber, the main minerals of alginate, such as calcium and magnesium, which are actively released in the soil, increase the hydration reaction in wet environments. Moreover, alginate fiber has the advantage of being biodegradable and renewable and is widely used in wastewater treatment, food processing, paper printing, textile dyeing and weaving, and other fields [32]. However, research studies and findings on stabilized river silt reinforced with cement and alginate fibers (AFCS) are limited. Hu et al. [32] analyzed the reinforcement mechanism of AFCS and established a correction prediction model for compressive strength, considering multiple parameters. The degradation behavior and mechanism of AFCS in different engineering environments are crucial for engineering applications. However, research studies on the degradation behavior and mechanism are sparse. Also, the degradation mechanism analysis of the composite mechanism of alginate fibers and cement has not been studied. Moreover, factors such as seawater erosion can affect the mechanical properties of AFCS when used in coastal areas. Hence, four engineering environments in coastal areas were included in this study, which were as follows: a drying and wetting environment, a freeze–thaw environment, an immersion environment and seawater erosion. Based on the above environments, freeze–thaw (F–T) cycle tests, wetting-drying (W–D) cycle tests, water immersion tests and seawater erosion tests were conducted to study the strength reduction mechanism. And the microstructure of AFCS was analyzed by scanning electron microscopy (SEM) imaging.

## 2. Materials and Experimental Methods

### 2.1. Materials

**Figure 1:**
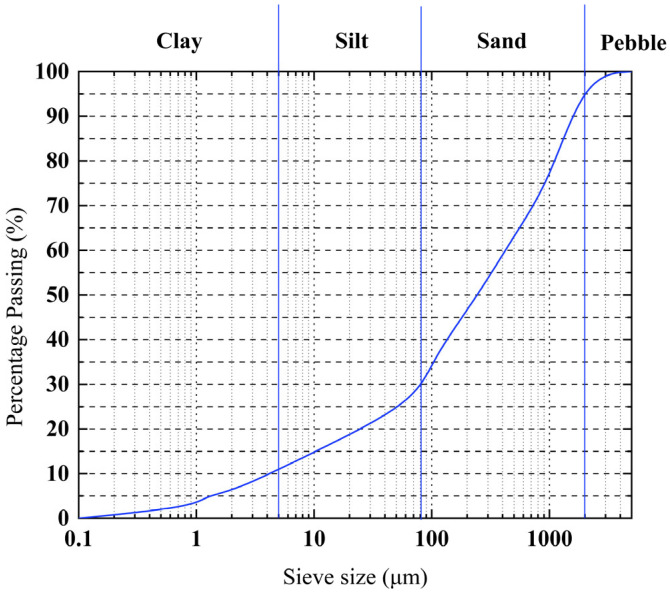
Cumulative particle size distribution of silt.

The river silt in the experiment was sediment of a river in Shandong Province, located in eastern China. The physical properties of the river silt determined by ASTM D2216-19 [33], ASTM D7263-21 [34], ASTM D4318-18 [35], ASTM D3080-04 [36], ASTM D854-14 [37] and ASTM D2487-17e1 [38] are presented in Table 1. Figure 1 shows the cumulative particle size distribution of the river silt. Since river silt stabilized with cement and reinforced with fibers is used as a bearing layer for in situ stabilization, the moisture content in this experiment should closely match that of field conditions. To attain a consistent moisture content in the experimental samples, the moisture content of all samples was set to the liquid limit.

**Figure 2:**
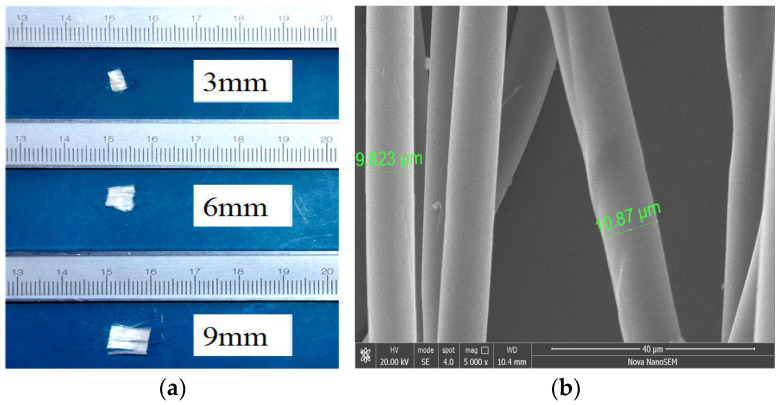
Alginate fibers for testing: (**a**) Alginate fibers; (**b**) SEM image of alginate fiber.

Alginate fibers, polypropylene fibers and ordinary Portland cement were applied as stabilizing agents to solidify the river silt. The macroscopic and microscopic morphology of alginate fibers is shown in Figure 2, and the physical characteristics are given in Table 2. The three lengths (3 mm, 6 mm and 9 mm) were applied to this study, and the average diameter of alginate fibers was 10 μm.

Cement (PO 42.5) produced by Anhui Conch Cement Co., Ltd. in Anhui Province, located in China. was used in this study; the chemical composition and physical properties on ignition of the cement are listed in Table 3.

### 2.2. Specimen Preparation

**Figure 3:**
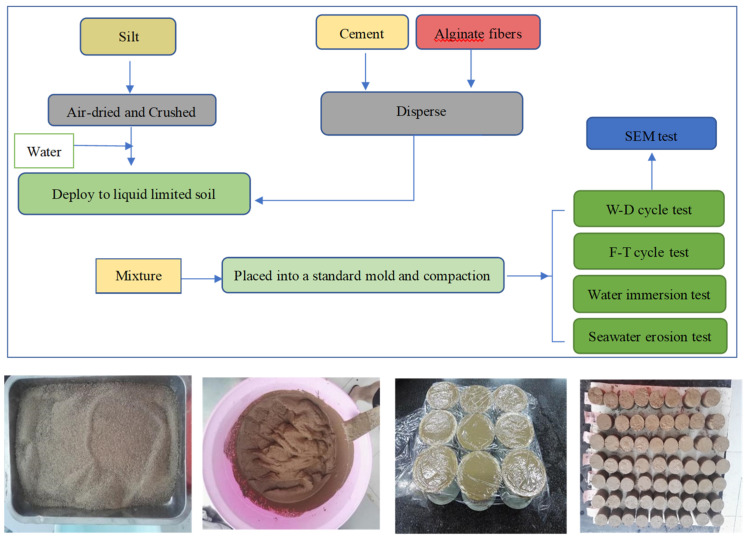
Experimental procedure.

Cylindrical specimens with 50 mm diameters and 50 mm heights were prepared in this study. The specimens were compacted for 5–10 s on a vibration table. The specimens were placed in a humidity chamber with a 20 ± 2 °C temperature and 95% relative humidity for 7 and 28 days. The specific experimental procedure is demonstrated in Figure 3. At the specified time, the specimens were subjected to a series of F–T cycle tests, W–D cycle tests, water immersion tests and seawater erosion tests. Scanning electron microscopy (SEM) tests were also conducted on the specimens.

### 2.3. Test Scheme and Methods

In order to analyze the strength degradation of the AFCS, the fiber content and length selected in each test were different. And cement-stabilized soil reinforced with polypropylene fiber (PFCS) was used as a comparative sample in the F–T cycle tests, indicating the properties of AFCS. The specific scheme is presented in Table 4, with three specimens prepared for each type as shown, and the main instruments and equipment used in the experiments are listed in Table 5. Hu et al. [32] confirmed that the strength of AFCS cured for more than 28 days did not significantly increase, and the strength with a cement content of 6% (by weight of wet soil, the same as below) was relatively lower (only 150 kPa). However, the strength with a cement content of 9% was 465 kPa at a curing time of 28 days. Therefore, in this study, a 28-day curing time and a 9% cement content were used, except for the seawater erosion tests, to analyze the degradation behavior and mechanism of AFCS with different fiber contents (0%, 0.3%, 0.6% and 0.9% by weight of wet soil) and fiber lengths (3 mm, 6 mm and 9 mm) in different engineering environments. Detailed procedures are presented in a later section.

#### 2.3.1. F–T Cycle Test

Three samples with a curing time of 28 days were prepared for the F–T cycle test. A freezing temperature of −15 °C was maintained for 12 h, followed by a thawing temperature of 15 °C for 12 h, completing one cycle of freezing and thawing. The number of F–T cycles was set to 0, 1, 3, 6, 10, 15, 20 and 25. After the number of F–T cycles was completed, the mass loss and strength of the specimens were measured.

Mass loss in the specimens occurred in the F–T cycle test. In order to analyze the mass loss in the cycle tests, the mass loss (ID) is expressed as in Equation (1).

(1) $$\mathrm{ID}=\frac{w_{0}-w_{n}}{w_{0}}\times 100\%$$

where ID is the mass loss (%) after n cycles of freezing and thawing; $w_{0}$ is the mass of the specimen before the test (g); and $w_{n}$ is the mass of the specimen after n cycles of freezing and thawing (g).

#### 2.3.2. W–D Cycle Test

Three specimens were prepared and cured for 28 days for the W–D cycle tests. The specimens were marked and placed in water for 24 h. After that, the specimens were placed in a ventilated laboratory for 24 h. This completed one wetting–drying cycle. The specimens were exposed to 3, 5, 10, 15, 20, 25 and 30 cycles to evaluate the change in the degradation behavior of AFCS. Last, the mass loss and UCS of the specimens were measured. In order to discuss the stability properties of the strength in the stabilized river silt after the W–D cycle tests, the stability coefficient is defined as in Equation (2).

(2) $$R_{s}=\frac{q_{b,n}}{q_{e}}$$

where $q_{b,n}$ is the average strength of the specimen after the W–D cycles and $q_{e}$ is the average strength before the W–D cycles.

#### 2.3.3. Water Immersion Tests

For the test procedure for water stability, the AFCS specimens, which were cured for the standard curing time of 28 days in a curing chamber at a standard constant temperature and humidity, were taken out, and their surfaces were wiped clean. Then, the specimens were immersed in water. If the water evaporated too quickly, it was added promptly so that the specimens remained submerged. Subsequently, the UCS tests were performed by immersing the specimens in water for 24 h. Finally, the water stability of the specimens was determined according to the immersed strength compared with the standard strength of the specimens. The softening coefficient was used to characterize the water stability of AFCS.

The formula for calculating the softening coefficient (γ) of AFCS is given in Equation (3).

(3) $$\gamma =\frac{f}{F}$$

where f is the UCS of specimens in a freshwater environment (kPa) and F is the UCS of specimens in a standard constant environment (kPa).

#### 2.3.4. Seawater Erosion Test

The seawater erosion test for AFCS was conducted following the following procedure: The prepared specimens were placed in curing chamber at a constant temperature and humidity for a curing time of 7 days. The specimens were immersed in seawater, and the upper surfaces of the specimens were about 2 cm below the liquid level. The specimens were cured in a seawater environment until the corresponding curing time. At the same time, the same proportion of specimens was cured in freshwater. Finally, the UCS was determined.

With the addition of alginate fiber, the UCS of AFCS was significantly improved. To further quantify the impact of the seawater erosion environment on the UCS of AFCS, the seawater erosion damage degree $D_{\mathrm{sea}}$ was determined, as defined in Equation (4).

(4) $$D_{\mathrm{sea}}=\left(1-\frac{{q_{n}}^{\prime }}{{q_{n}}^{\prime \prime }}\right)\times 100\%$$

where $D_{\mathrm{sea}}$ is the seawater erosion damage degree; ${q_{n}}^{\prime }$ is the UCS after n days of curing in a seawater environment (kPa); and ${q_{n}}^{\prime \prime }$ is the UCS after n days of curing in a freshwater environment (kPa).

#### 2.3.5. SEM Imaging

A Nova NanoSEM450 scanning electron microscope was used for SEM imaging. AFCS specimens were taken from the fractured samples, and the test procedure for SEM imaging followed by Wang et al. [44] was used for the research specimens with a curing time of 28 days. The specimens consisted of AFCS with different contents and lengths of alginate fiber, as well as AFCS after immersion and AFCS after freezing and thawing.

## 3. Test Results and Discussion

### 3.1. F–T Cycle Test

**Figure 4:**
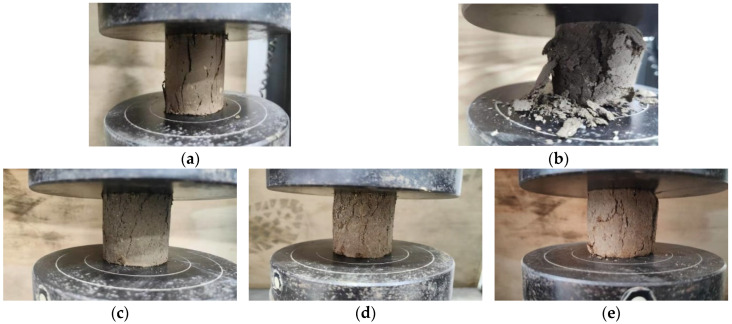
Failure specimen in F–T cycle test: (**a**) CS after 0 F–T cycles; (**b**) CS after 15 F–T cycles; (**c**) AFCS after 0 F–T cycles; (**d**) AFCS after 15 F–T cycles; (**e**) AFCS after 25 F–T cycles.

The surface fractures of the samples shown in Figure 4 were taken when the UCS of the specimen reached its peak. Since the specimens of CS were completely broken at 20 F–T cycles, only the surfaces of the specimens with 0 and 15 F–T cycles were captured. Figure 4a,b show that the specimen fragmentation degree of CS specimens intensified with F–T cycles. Before the freeze–thaw cycles, only obvious cracks appeared under the load, and significant peeling occurred under the load after the F–T cycles. Figure 4c–e indicate that cracks in the AFCS specimens generated under the load increased with the increasing number of F–T cycles. At the 25th F–T cycle, significant cracking was observed. It can also be seen from Figure 4a–d that the number of cracks generated and the degree of specimen fragmentation of the AFCS specimens were lower than those of the CS specimens under a load when the F–T cycles were the same.

**Figure 5:**
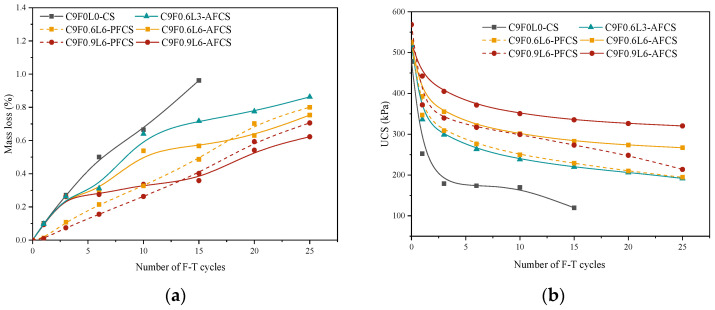
Physical properties and strength of samples after F–T cycle test: (**a**) ID; (**b**) UCS.

The strength degradation properties of AFCS were investigated in extreme cold climatic conditions achieved by subjecting 28-day cured specimens to 25 F–T cycles. The mass loss (ID) of AFCS, PFCS and CS after 0, 1, 3, 6, 10, 15, 20 and 25 F–T cycles is shown in Figure 5a. The results show that the ID increased with the number of F–T cycles. All the specimens showed the same mass loss rate at the first F–T cycle. The same ID at the first F–T cycle is probably due to the removal of damaged bonded clay particles [45]. After that, the ID of CS exhibited a linearly increasing trend with the number of F–T cycles. However, the ID increase in AFCS and PFCS gradually diminished with the increasing number of F–T cycles. It was also observed that the ID of AFCS and PFCS showed lesser mass loss than CS. For the ID of AFCS and PFCS, the ID of AFCS was more than that of PFCS in the initial phase (before the 15th F–T cycle); however, the ID of AFCS was less than that of PFCS in the later stage (after the 15th F–T cycle). The reason for this is that polypropylene fiber has good dispersion in the beginning stage, owing to its large diameter and small density. Simultaneously, polypropylene fibers possess the characteristics of high tensile strength and elasticity, which increase the spatial constraint of fiber on the cement-stabilized soil, resulting in a small mass loss in PFCS. Moreover, the number of alginate fibers was larger, owing to the smaller diameter at the same fiber content and length; hence, the friction between the alginate fiber and soil particles reduced the ID of AFCS with the development of internal cracks of CS in the later stage. This also suggests that AFCS has long-term stability.

The ID of AFCS was increased from 2.89% to 62.71% as the F–T cycle number increased from 2 to 15 compared to CS, and the ID of AFCS was reduced with the increase in the length and content of alginate fibers. Fibers play a role in this phenomenon by providing more contact area for soil particles. Moreover, the rough surface of the fibers enhances interparticle cohesion from a physical perspective [20], increasing the soil particle resistance against detaching from the AFCS surface. Therefore, the overall mass loss of AFCS is reduced.

Figure 5b shows the strength of specimens subjected to 0, 1, 3, 6, 10, 15, 20 and 25 F–T cycles. The UCS of specimens reduced with the increasing F–T cycles because the free water and bound water underwent continuous freezing and melting as the F–T cycles proceeded [46], leading to microcrack generation within the samples. Additionally, the bonding of C-S-H gel was also disrupted [5], resulting in a macroscopic reduction in UCS and eventual failure of the samples after a certain number of F–T cycles.

After 1, 6, 10 and 15 F–T cycles, the strength of CS decreased from 478.22 kPa to 252.51 kPa, 173.66 kPa, 169.62 kPa and 119.65 kPa, respectively. For AFCS, taking an alginate fiber content of 0.6% and a length of 6 mm as an example, the strength of AFCS reduced from 521.29 kPa to 392.86 kPa, 321.51 kPa, 301.14 kPa and 282.52 kPa. The strength of PFCS reduced from 525.55 kPa to 347.13 kPa, 276.11 kPa, 249.32 kPa and 228.76 kPa. The above results show that the strength loss was significant after the first F–T cycle for all stabilized soils, especially for CS due to the use of a small amount of cement (9%), which is consistent with the results of Sahlabadi et al. [22] and Boz et al. [47] for small amounts of cement or lime. And the strength loss rate of AFCS was lower than that of CS with the addition of fibers. So, the fibers could exert constraints on the many gaps and macro-fractures formed by the freeze–thaw engineering environment.

For the UCS of AFCS and CS, the UCS of AFCS was higher than that of CS for the same number of F–T cycles, and the difference between them increased with the increase in F–T cycles. And the UCS of AFCS improved from 56% to 136% as the F–T cycle number increased from 1 to 15 compared to CS. It can be further observed in Figure 5b that the maximum and minimum decrement values in the UCS of AFCS were 215.52 kPa and 99.96 kPa compared with the UCS of CS, corresponding to a fiber length of 6mm with a 0.9% content and a fiber length of 3 mm with a 0.6% content. The UCS of AFCS was improved by 84% and 180% at 15 F–T cycles. The results also indicate clearly that the decrement values increased with the length and content of fibers. And the strength of AFCS was improved with the increase in the length and content of alginate fibers. This is attributed to the spatial network structure formation and bridging effect of the fibers within the samples [20], enhancing the UCS of AFCS and inhibiting crack propagation in the soil. As a result, the decrease in the UCS of AFCS was mitigated. Further, the tensile effect of the fibers [4] helped maintain the structural integrity of AFCS under the influence of F–T cycles, preventing extensive cracking and even failure. The F–T cycle test revealed that adding alginate fiber significantly improved the quality and strength of the stabilized soil.

For the UCS of AFCS and PFCS, at the same fiber content and length, the UCS of PFCS was higher than that of AFCS before the F–T cycle test due to the larger tensile strength of polypropylene fiber. However, the UCS of PFCS was less than that of AFCS after the first freeze–thaw cycle, which may have been due to the larger number of alginate fibers at the same fiber content and length, resulting in stronger friction between the alginate fibers and soil particles with the development of internal cracks in cement-stabilized soil.

### 3.2. W–D Cycle Test

**Figure 6:**
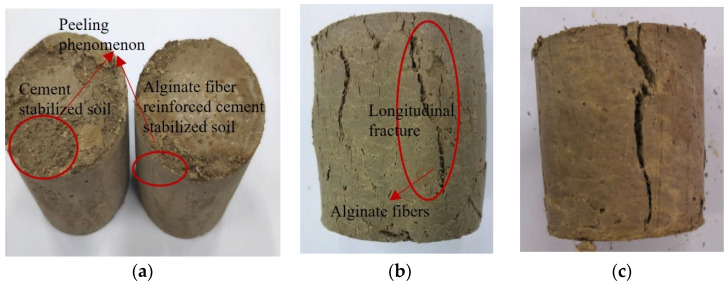
Failure specimen for W–D cycle test: (**a**) Before UCS test; (**b**) After UCS test with 5 W–D cycles; (**c**) After UCS test with 30 W–D cycles.

There are rainy and dry seasons in northern China. The shrinkage of foundation soil may be caused by water loss in the autumn and winter seasons, and the swelling of foundation soil is caused by water absorption in the spring and summer seasons. Irreversible changes in the structure of the foundation soil are attributed to the shrinkage or swelling of the foundation, decreasing the soil strength and leading to foundation damage. Hence, the strength deterioration of stabilized soil with alternative wetting and drying in water currents was studied by subjecting 28-day cured specimens to 30 W–D cycles. Figure 6a shows that the peeling phenomenon of the upper surface appeared, and the peeling phenomenon of AFCS was reduced compared with that of CS. However, there was no evident peeling phenomenon of the axial surface layer in the AFCS, indicating that this may have been due to the reinforcing effect of the internal alginate fibers in the specimen. Moreover, a longitudinal fracture appeared on the surface of the specimen after conducting the strength test, as shown in Figure 6b,c. The alginate fibers can be seen in the longitudinal fracture. Also, the length and depth of the cracks increased with the number of W–D cycles.

**Figure 7:**
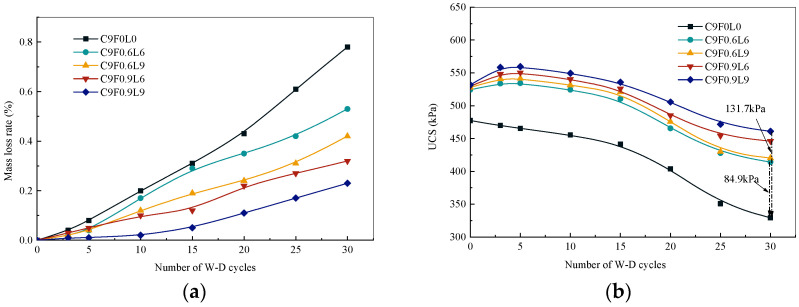
Physical properties and strength of samples after W–D cycle test: (**a**) ID; (**b**) UCS.

The variation pattern between the mass loss (ID) and the number of W–D cycles is plotted in Figure 7a. It can be observed that the ID gradually increased with the increasing number of W–D cycles, and the ID of AFCS was lower than that of CS. Moreover, the ID was reduced with the increased length and content of alginate fibers. The ID of AFCS was reduced between 32% and 71% when the fiber content increased from 0.6% to 0.9% and the fiber length increased from 6 mm to 9 mm compared with that of CS at 30 W–D cycles. This is because the structure of the cement hydration products was destroyed with the increasing number of W–D cycles [22]. Additionally, the overall integrity of AFCS was enhanced by adding alginate fibers [48]. Consequently, the surface peeling phenomenon and the ID of AFCS were significantly reduced compared to CS. Further, it was noticed that the ID of AFCS reduced with the increase in fiber content and length, attributed to the similar plant root mechanism of alginate fibers to reinforce silt [49]. Thus, the pores in CS were filled by fibers, forming a more stable and firm structure within the AFCS. Moreover, the frictional force occurred at the contact area between the alginate fiber and the stabilized particles by subjecting the lateral load, suppressing soil deformation [50].

The maximum mass loss was 0.8% for all specimens in the W–D cycle test, whereas in the F–T cycle test, the maximum mass loss was 1.0%, indicating good stability against wetting–drying compared to freezing–thawing. The permissible limit of mass loss is 14% according to the Portland Cement Association (PCA) for cement-stabilized granular soils after 12 cycles of W–D and F–T [49,50,51]. AFCS showed excellent stability against wetting–drying and freezing–thawing impacts on mass loss.

The resistance of AFCS against strength loss when exposed to W–D cycle tests was stronger than that of CS, as shown in Figure 7b. After 5, 10, 15 and 20, 30 W–D cycles, the strength of CS decreased from 478.22 kPa to 465.36 kPa, 455.36 kPa, 441.49 kPa, 403.73 kPa and 329.21 kPa, respectively. For AFCS, taking an alginate fiber content of 0.6% and a length of 6 mm as an example, the strength of AFCS was reduced from 521.29 kPa to 534.31 kPa, 521.31 kPa, 510.42 kPa, 465.59 KPa and 414.15 kPa. The UCS of AFCS was higher than that of CS for the same number of W–D cycles. The UCS of AFCS improved from 10% to 26% as the W–D cycle number increased from 0 to 30 compared to CS. It was further observed that the UCS ratio of AFCS to CS increased with the increasing W–D cycles, especially in the later stage of W–D cycle tests. At 30 wetting–drying cycles, the maximum and minimum decrement values in UCS were 131.7 kPa and 84.9 kPa, corresponding to a fiber length of 9 mm with a 0.9% content and a fiber length of 6mm with a 0.6% content. Compared with the UCS of CS, the UCS of AFCS was improved by 26% and 40% at 30 wetting–drying cycles. Also, the UCS improved with the increased fiber length and content of alginate fibers. The influence of W–D cycles on the UCS of samples with added alginate fibers was reduced, and this trend was more pronounced with an increase in alginate fiber content. This was attributed to microcrack appearances in the weak spots of the soil due to continuous wet swelling and dry shrinkage in AFCS during the W–D cycle test. Thus, the AFCS strength was reduced. However, crack development was suppressed effectively by the addition of alginate fibers. So, the deformation of AFCS was reduced, and the adverse effects of W–D cycles on the strength of AFCS were also reduced [4,52].

With the increase in W–D cycles, the UCS of CS decreased gradually; nevertheless, the UCS of AFCS increased first slightly and then decreased. During the early stage of W–D cycles (within three cycles), the reason for the increased UCS of AFCS was that the hygroscopic characteristics of alginate fiber could be exerted by contact with the stabilized soil in the wet environment. Then, the main minerals such as calcium and magnesium of alginate actively released in the stabilized soil, which was also verified from the element proportion, as shown in Table 6. After that, the hydration reaction of cement could be increased in the dry environment. So the strength, which was improved by cement hydration reactions in AFCS, was greater than the negative impact of W–D cycles [22]. In the middle stage of wetting–drying cycles (3–5 cycles), a peak strength appeared in the UCS of AFCS, and the strength development rate was 2.0% to 8.0%. During the later stage of the wetting–drying cycles (more than five cycles), the UCS of AFCS was reduced with increasing W–D cycles, which was attributed to the damaged structure by the repeated cycles and voids created in the soil by water migration [1]. When the number of W–D cycles reached 30, the strength decreased by 13.31% to 21%, validating that the resistance strength loss ability of AFCS was improved.

From the obtained results, it can be deduced that the strength degradation of AFCS after 30 W–D cycles was lower than that of CS, signifying enhanced resistance of the stabilized soil with the alginate fiber addition. Also, the lifespan of the specimen was extended with the standard required strength, providing a reference for the service life evaluation of projects situated in areas with seasonal precipitation.

### 3.3. Residual Strength Index Model of W–D Cycles

**Figure 8:**
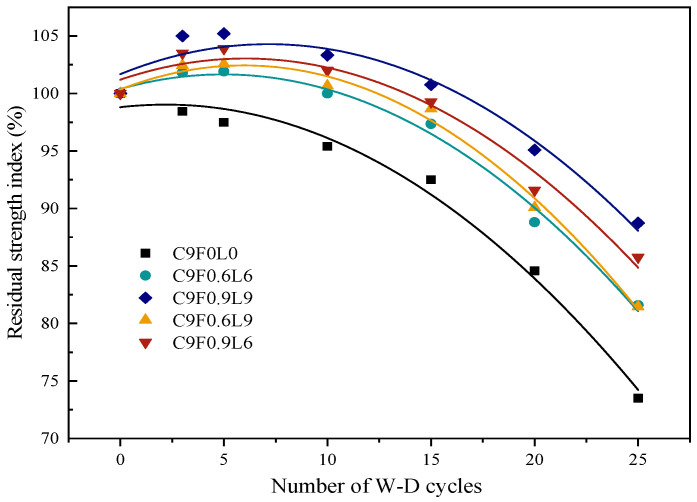
The residual strength index of AFCS and the number of W–D cycles.

The variation in the residual strength index of AFCS versus the number of W–D cycles is plotted in Figure 8.

The variation trend in the residual strength index with the number of W–D cycles can be expressed as a quadratic function according to Equation (5).

(5) $$R_{s}=a\times N^{2}+b\times N+c$$

where $R_{s}$ is the residual strength index; N is the number of W–D cycles; and a, b and c are the parameters.

The parameter values of a, b and c for different contents and lengths of alginate fibers are given in Table 7.

Table 7 indicates the values of parameter a changed a little with the increase in the length and content of alginate fibers. Therefore, the average value of 0.05184 was taken as the final parameter a. It was further observed that the value of parameter c showed significant variation with the alginate fiber content, whereas it showed a minor change with the alginate fiber length. Hence, a linear relationship between the variation in parameter c and the alginate fiber content was obtained, which is given in Equation (6).

(6) c=2.8833×F+98.763

The effect of the alginate fiber content and length on parameter b is given in Equation (7).

(7) $$b=-0.58996+0.79719\times e^{\frac{F}{6.12184}}\times e^{\frac{L}{24.42469}}$$

In order to quantitatively evaluate the effects of the alginate fiber content, fiber length and number of W–D cycles on the residual strength index of AFCS, Equations (6) and (7) were substituted into Equation (5), and Equation (8) was obtained.

(8) $$R_{s}=-0.05184\times N^{2}+\left(-0.58996+0.79719\times e^{\frac{F}{6.12184}}\times e^{\frac{L}{24.42469}}\right)\times N+2.8833\times F+98.763$$

where R_s_ is the residual strength index of AFCS; N is the number of W–D cycles; F is the alginate fiber content; and L is the alginate fiber length.

**Figure 9:**
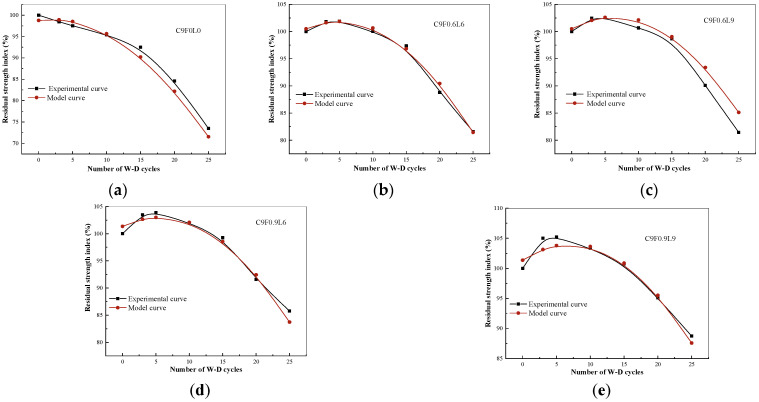
Compared the experimental curve with the model curve: (**a**) C9F0L0; (**b**) C9F0.6L6; (**c**) C9F0.6L9; (**d**) C9F0.9L6; and (**e**) C9F0.9L9.

The comparison of the model and the experiment shown in Figure 9 clarifies that the experimental data matched well with the model established in this study. The correlation coefficients for each group were above 0.95, indicating a better relationship between the residual strength index and the number of W–D cycles, as well as the fiber content and length.

### 3.4. Water Immersion Test

The stability of specimens (for 28-day cured specimens) in the water immersion was evaluated based on a heavy rainstorm. The softening coefficient is a vital indicator characterizing the water stability performance, and its standardized value is between 0 and 1. The closer the value of the softening coefficient to 1, the higher the water resistance of the AFCS. Likewise, the closer the value of the softening coefficient to 0, the lower the water resistance of the AFCS. According to current national regulations [53,54], the softening coefficient of the material must not be less than 0.85 for essential buildings that have been in damp or water environments for a long time, and the softening coefficient must not be less than 0.70 for relatively humid or minor buildings. Generally, materials with softening coefficient values > 0.85 are considered water-resistant by the engineering community. The immersed and standard UCS and softening coefficients of AFCS are presented in Table 8. The softening coefficients of AFCS exceeded 0.85, except for that with an alginate fiber content of 0.6% and an alginate fiber length of 3 mm, which indicated better water stability. Therefore, AFCS can be considered a water-resistant material, meeting the deterioration requirements for material in road construction. The value lower than 0.85 may have been because the alginate fiber was not evenly dispersed in the soil during specimen preparation. With the increase in the length and content of alginate fibers, the softening coefficients of AFCS were improved in the water immersion conditions, indicating lower strength degradation of AFCS.

**Figure 10:**
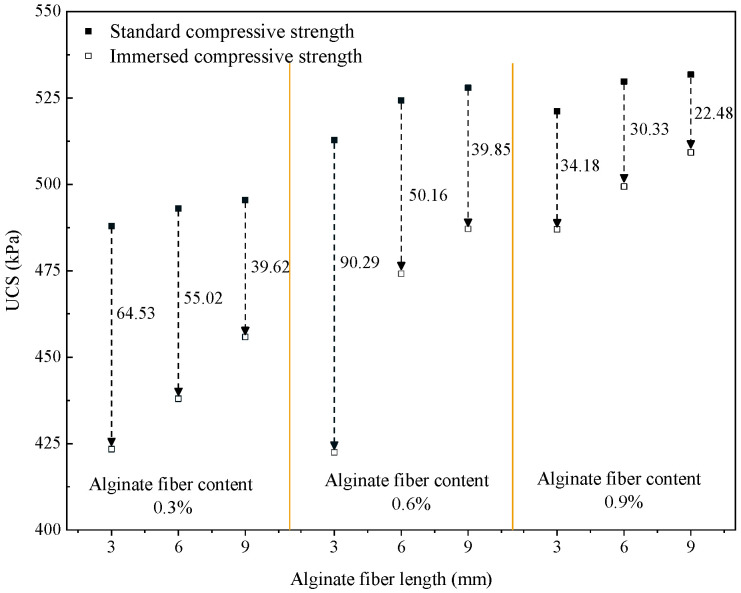
Influence on the strength of AFCS for both standard and immersion conditions.

To further quantitatively evaluate the effect of moisture intrusion on the strength, the UCS values for both standard and immersion conditions were determined, as shown in Figure 10. Compared to the standard curing environment, the UCS of AFCS significantly decreased under the immersion environment. Except for the AFCS with an alginate fiber content of 0.6% and alginate fiber length of 3 mm, the maximum strength reduction was 64.53 kPa, which was found in that with an alginate fiber content of 0.3% and an alginate fiber length of 3 mm. With the increase in the length and number of alginate fibers, the UCS of AFCS was improved, and the minimum reduction in UCS was 22.48 kPa for the samples with alginate fiber contents of 0.9% and alginate fiber lengths of 9 mm. It has been shown earlier that strength deteriorates in an immersion test for AFCS mainly because the bonding between soil particles is weakened due to water immersion [1,55], reducing cohesion between soil particles. Also, water absorption and swelling in river silt causes rapid expansion in the volume of the samples after water immersion, leading to increased porosity and irregular deformation between internal soil particles. Microcracks occur by the stress concentration and damage the internal structure [1]. The aforementioned factors contributed to the damage to the internal structure of the sample, loosening it and resulting in decreased UCS.

### 3.5. Seawater Erosion Test

**Figure 11:**
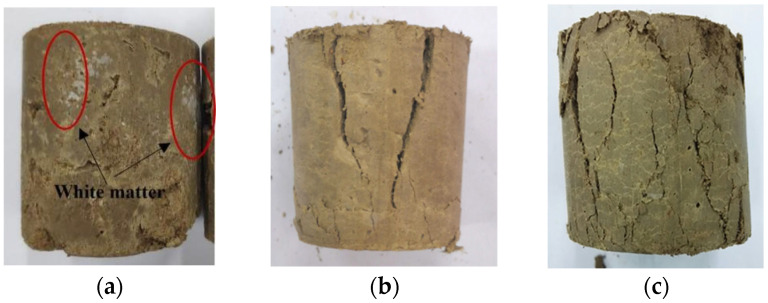
Failure pattern diagram of specimens: (**a**) Before UCS test; (**b**) After UCS test in the freshwater environment; (**c**) After UCS test in the seawater environment.

The effects of seawater immersion on the strength and degradation properties of specimens were evaluated by immersing specimens with a 7-day curing time for 7, 14, 28 and 60 days in a seawater environment. Figure 11a shows that the specimens cured in the seawater environment before the UCS test were coated with a white material on the surface, which was also produced as a precipitate on the bottom of the solution. With the increase in the erosion time, some test specimens gradually fell off. The surface peeling phenomenon for the specimens cured in the seawater environment after the UCS test was prominent, as shown in Figure 11b,c. The surface erosion of the specimens in the seawater environment was higher than that of the other specimens immersed in freshwater.

**Figure 12:**
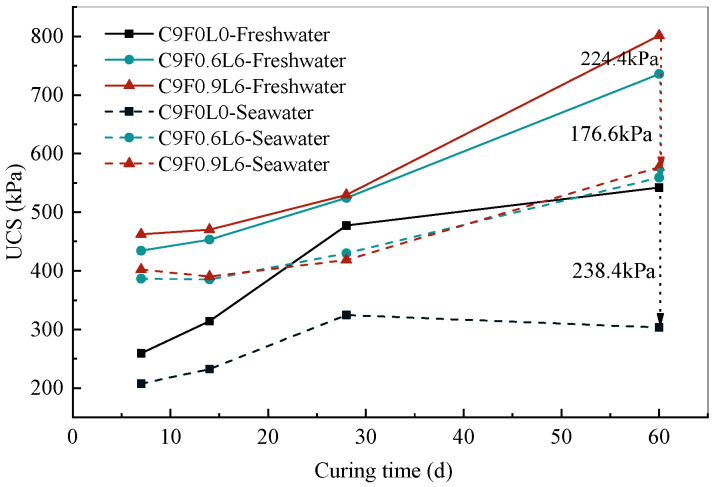
UCS with curing time under seawater and freshwater environments.

The change in the UCS with curing time under seawater and freshwater environments is shown in Figure 12. It is noted that the UCS of AFCS was higher than that of CS in the same environment, and the UCS of specimens in the freshwater environment was higher than that in the seawater environment with the same fiber content. Moreover, the strength of CS in the seawater environment was reduced to 20–32% compared with that in the freshwater environment, and the strength of AFCS was reduced to 11–21%. Therefore, the strength of AFCS was improved about 10% compared with that of CS. The analysis of the UCS of specimens with the same fiber length of 6 mm cured in seawater and freshwater environments also indicated lower UCS reductions with increasing curing times. The maximum reduction in UCS was 238.4 kPa, which was found in the CS. The reduction in the UCS of AFCS was improved with the increase in the length and content of alginate fibers, and the minimum decrement in UCS was 176.6 kPa, which was found in AFCS with an alginate fiber content of 0.6%, indicating that the optimal fiber content was 0.6%. This conclusion is consistent with previous findings [32]. It was also observed that the UCS of AFCS increased slowly before the 28-day curing time, and UCS development in CS was greater than that in AFCS. However, the UCS development rate of AFCS increased significantly when the curing time exceeded 28 days. This illustrates that alginate fiber could help enhance the resistance of stabilized soil against seawater erosion.

**Figure 13:**
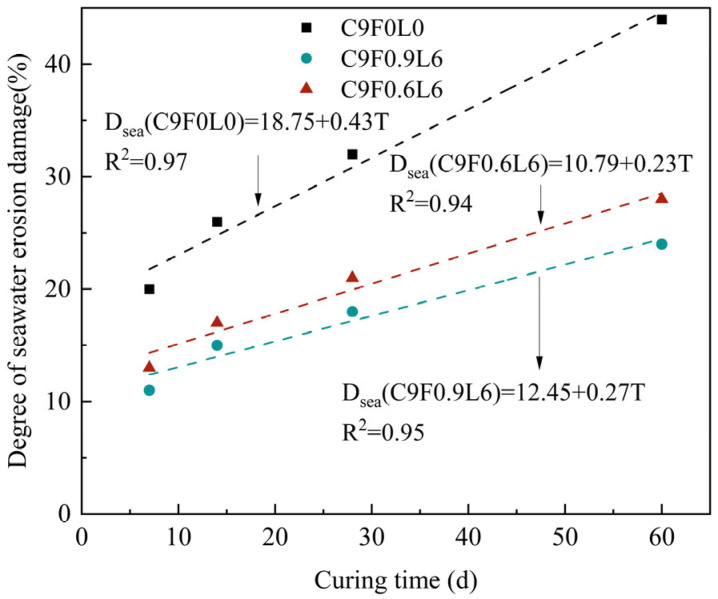
Seawater erosion damage degree with curing time in seawater environment.

The degree of erosion damage in the seawater environment was obtained by Equation (4), and the results are shown in Figure 13. It can be observed that the seawater erosion damage degree of CS was greater than that of AFCS for the same curing time. Further, the degree of erosion damage of AFCS in the seawater environment linearly increased with the increase in curing time and fiber content. The rate of the seawater erosion damage degree in AFCS decreased compared with CS because of the chemical composition of ordinary Portland cement (${\mathrm{CaO},\mathrm{SiO}}_{2}{,\mathrm{Al}}_{2}O_{3}{,\mathrm{Fe}}_{2}O_{3}{,\mathrm{SO}}_{2}$). The primary ions eroded by seawater are ${\mathrm{Mg}}^{2+}$, ${\mathrm{Cl}}^{-}$ and ${{\mathrm{SO}}_{4}}^{2-}$. First, ${\mathrm{Mg}}^{2+}$ ions reacted with cement soil to form $\mathrm{MgO}\cdot {\mathrm{SiO}}_{2}\cdot H_{2}O$, and $3\mathrm{CaO}\cdot {\mathrm{SiO}}_{2}\cdot {2H}_{2}O$ was dispersed, negatively impacting the cementitious properties. Thus, the strength of cement soil was reduced at the macro level. Then, the expansion effect occurred due to ${\mathrm{CaSO}}_{4}\cdot 2H_{2}O$ generated by the cement soil and ${{\mathrm{SO}}_{4}}^{2-}$ ions. With the increase in the ${{\mathrm{SO}}_{4}}^{2-}$ content, when the expansion force was greater than the bonding force of the cementitious soil, the strength decreased at the macro level as the cementitious soil failed. In addition, the degree of erosion (e.g., peeling, cracking and swelling) varied due to differences in influencing factors, such as soil quality, seawater erosion time and seawater pressure, which resulted in reduced overall integrity of the cement soil and a lower bearing capacity.

Under seawater exposure, erosion gradually progressed from the surface toward the inside as the seawater immersion time prolonged (the curing time increased). The addition of alginate fibers delayed the erosion depth of AFCS, as the fibers enhanced the tensile strength of the AFCS. River silt particles were consolidated by the three-dimensional mesh skeleton of fibers, so the AFCS’s strength could be maintained under various salt conditions. This indicates that the fibers could resist the erosion and damage of the sample in the seawater environment.

### 3.6. SEM Imaging

The main mechanism of cement-enhanced AFCS is the cement hydration reaction, which forms hydration products. The cement hydration reaction is a process accompanied by a significant release of heat and a series of chemical reactions. This process is mainly driven by the interactions of tricalcium silicate and dicalcium silicate in cement. The reaction can be described by a chemical equation:

At room temperature, calcium silicate hydrate (C-S-H gel) and calcium hydroxide are produced by the hydration reaction of tricalcium silicate.

(9) $$3CaO\cdot SiO_{2}+nH_{2}O=xCaO\cdot SiO_{2}\cdot yH_{2}O+\left(3-x\right)Ca{\left(OH\right)}_{2}$$

The hydration reaction principle of dicalcium silicate is similar to that of tricalcium silicate, except that the hydration rate is slightly slower than that of tricalcium silicate.

(10) $$2CaO\cdot SiO_{2}+nH_{2}O=xCaO\cdot SiO_{2}\cdot yH_{2}O+\left(2-x\right)Ca{\left(OH\right)}_{2}$$

**Figure 14:**
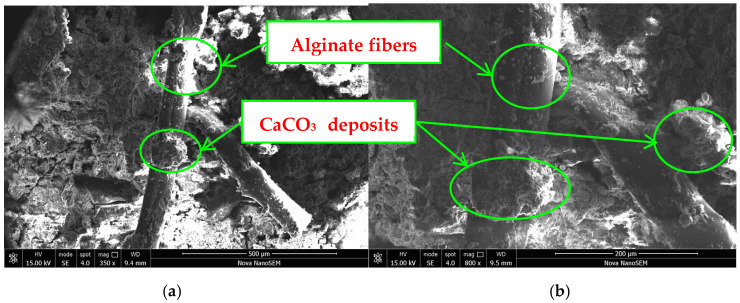
SEM image of the bonding interface between alginate fiber and CS: (**a**) ×350; (**b**) ×800.

**Figure 15:**
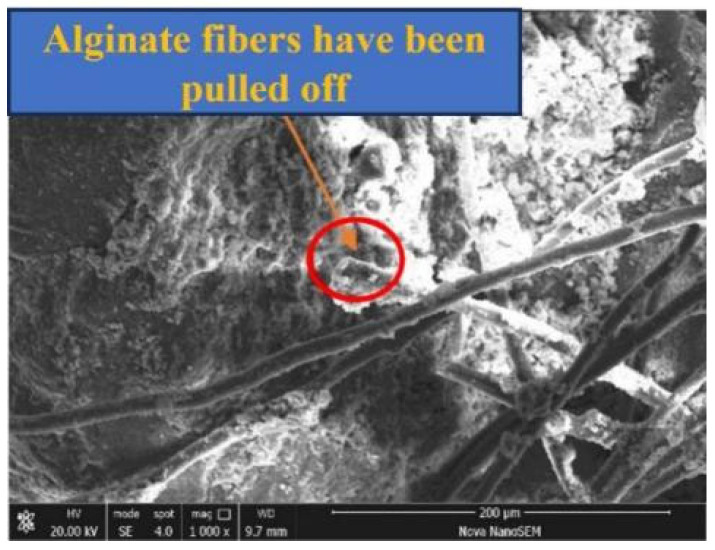
Microscopic morphology of AFCS.

The hydration products formed by the hydration reactions of tricalcium silicate and dicalcium silicate, namely calcium silicate hydrate, are presented in Figure 14 and Figure 15. The calcium silicate hydrate agglomerates two or more loose silt particles into an aggregate and fills pores between silt particles, cementing with each other to form a larger coacervate. The presence of C-S-H gel can enhance the strength of AFCS and the density of its internal structure.

**Figure 16:**
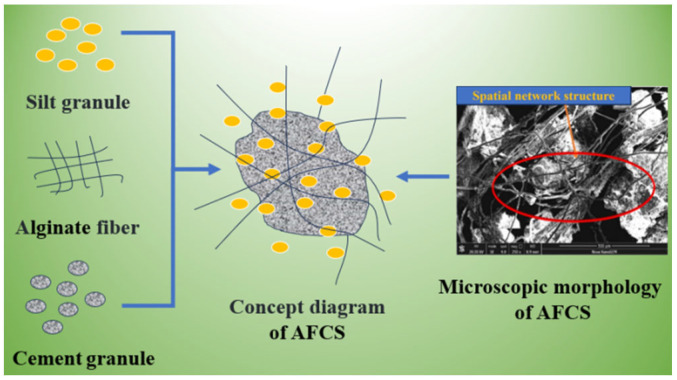
Concept diagram and micrograph illustrating the interaction of AFCS.

The main mechanism by which alginate fibers enhance AFCS is the bending and intertwining of alginate fibers. A concept diagram and micrograph illustrating the interaction of AFCS are shown in Figure 16 and the SEM imaging of alginate fibers are also presented in Figure 14 and Figure 15 The alginate fibers were twisted and interlaced between the river silt particles and cement hydrates. Simultaneously, cement hydration products (C-S-H and portlandite) were attached to the alginate fiber surface, forming larger tree-like aggregations. This confirmed strong physical and chemical adhesion, owing to better interfacial bonding between alginate fibers and CS. Figure 16 shows that the alginate fibers in the field of view were intertwined with each other and distributed evenly, forming a spatial network structure due to the bridging effect of twisting and interlacing [56,57]. River silt particles adhered to the fiber surface, and alginate fibers were wrapped by river silt particles and cement hydrates. Hence, the spatial movement and deformation of river silt particles were restricted and constrained by strong bonding and a frictional force between alginate fibers and river silt particles. This led to strength and stability enhancement of the river silt. In Figure 15 the end of the alginate fiber is visible, indicating that the alginate fibers were pulled off due to fiber inability to resist the tension generated.

**Figure 17:**
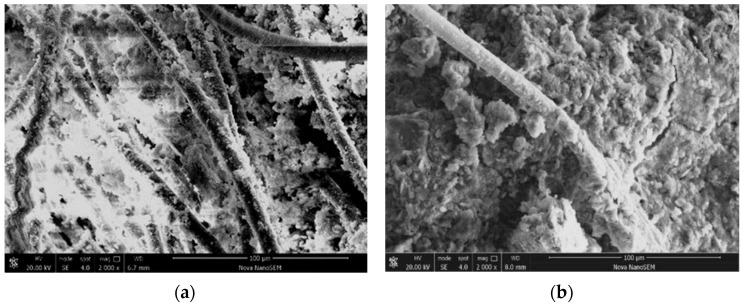
SEM images of fiber-reinforced cement-stabilized soil in the standard and immersed environment: (**a**) Standard samples; (**b**) Immersed samples.

Figure 17a,b exhibit that larger volumes comprised of soil particles and C-S-H gel were revealed in the immersion specimens compared with the standard specimens (Figure 17a,b show that, compared to the standard specimens, the immersed specimens exhibited significant cracks, which were caused by the expansion of soil particle volumes.). Thus, these aggregates needed more C-S-H gel to encapsulate and connect. In this scenario, some weak internal structures within the specimens were developed because some aggregates failed to contact with each other. Hence, these weak structures caused the failure and deformation of specimens. Also, the close connection of aggregates in the standard specimens signified the formation of a denser structure. The micro-morphological features of AFCS with better integrity and denser structures are shown in Figure 17a, indicating no visible cracks [58]. Additionally, the cement hydration products adhered to the surface of alginate fibers mixed with river silt particles. During AFCS curing, the squeezing state of the alginate fibers was generated by expansion and compaction caused by the cement hydration reaction; thus, the bite effect and the friction coefficient of the interface between alginate particles and river silt particles were enhanced, confirming that the frictional resistance of the interface between alginate fibers and soil particles was improved.

Noticeable cracks were found in the immersion specimens, as shown in Figure 17b. Visible microcracks in AFCS could be seen, indicating the damaged soil structure. With the external load, microcracks were produced due to the reduced bonding force between the alginate fiber and the river silt particles. With the increase in the external load, more cracks were produced in the surroundings, which gradually developed into large or through cracks. The stabilized soil was categorized based on the size and shape of vertical and horizontal cracks. Consequently, the “surface–surface” contact way gradually turned into the “point–surface” contact way and then into the “point-point” contact way, ultimately damaging the structure and integrity of CS. These cracks increased the deformation of AFCS.

**Figure 18:**
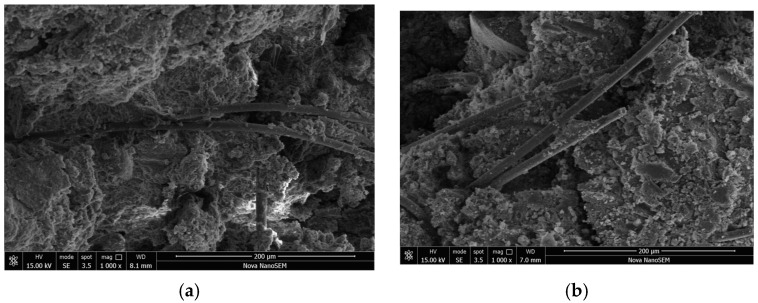
SEM images of fiber-reinforced cement-stabilized soil before and after F–T cycling: (**a**) Before F–T cycling; (**b**) After F–T cycling.

The microstructures of AFCS before and after F–T cycling are shown in Figure 18a,b. It can be observed in Figure 18a that the overall structure of AFCS was relatively compact before F–T cycling. The soil particles, C-S-H gel and alginate fibers were closely bonded together, enhancing the integrity of the AFCS structure. The surfaces of the agglomerates composed of soil particles and C-S-H gel were relatively intact and rounded, and the alginate fibers interwove inside the AFCS, providing better tensile strength. Whereas in Figure 18b, it can be found that significant cracks developed within the AFCS after F–T cycling, indicating a loss of structural integrity and increasing susceptibility to deformation under a load. Additionally, the surfaces of the agglomerates composed of soil particles and C-S-H gel became rougher due to the damaging effects of the freeze–thaw cycles on the C-S-H gel. Some of the C-S-H gel could not maintain its original form, and some soil particles were exposed due to the detachment of the C-S-H gel. This disruption of the bond between the C-S-H gel and soil particles reduced the load-bearing capacity of the AFCS. Furthermore, Figure 18b also reveals that some of the alginate fibers within the AFCS fractured after F–T cycling, disrupting the stable structure formed by the interaction of the fibers. These decreased the fiber’s ability to resist specimen deformation.

It can be deduced from the results that uniformly dispersed alginate fibers in CS improve strength. Through the deterioration tests and microscopic morphology, it can be observed that alginate fibers can enhance the mechanical properties of AFCS. When alginate fibers were subjected to tension in the soil, the resistance to relative sliding was improved effectively by the increased interface friction between alginate fibers and river silt particles, thus improving the tensile and compressive strengths of AFCS.

## 4. Conclusions

A series of tests were performed to investigate the strength degradation and microstructure evolution of AFCS. The main conclusions drawn from the obtained results are as follows.

Freeze–thaw (F–T) cycles substantially affect AFCS, PFCS and CS. The ID of AFCS was more than that of PFCS in the initial phase (before the 15th F–T cycle); however, the ID of AFCS was less than that of PFCS in the later stage (after the 15th F–T cycle). At the same fiber content and length, the UCS of PFCS was higher than that of AFCS before the F–T cycle test due to the larger tensile strength of polypropylene fiber. However, the UCS of PFCS was less than that of AFCS after the first F–T cycle. Moreover, the ID of AFCS was reduced from 2.89% to 62.71% compared to CS, and the UCS of AFCS improved from 84% to 180% at 15 F–T cycles. The decrement values increased with the length and content of fibers, which shows that AFCS exhibited significantly better performance in the F–T cycle test.

The wetting–drying (W–D) resistance and UCS of AFCS were significantly better than those of CS. The mass loss (ID) of AFCS was reduced between 32% and 71%, and the UCS of AFCS improved by 26% and 40% at 30 wetting–drying cycles. The stability coefficient can also be expressed as a quadratic function relationship with the number of W–D cycles, fiber content and fiber length. The correlation coefficients for each group were above 0.95, indicating the model’s reliability.

The UCS of specimens in the freshwater environment was higher than that in the seawater environment with the same fiber content. And the softening coefficient of AFCS in the freshwater environment was above 0.85, indicating that the AFCS had good water stability and could meet the requirements of material deterioration in road construction. The optimal fiber content was determined as 0.6% based on the analysis of the UCS reduction in cured specimens under seawater and freshwater environments. The strength of AFCS improved by about 10% compared with that of CS in the seawater environment. This indicates that alginate fiber could resist seawater erosion and prevent damage to the samples.

A spatial mesh structure of “alginate fiber–cementation products–river silt particles” is formed by the three components being tightly combined, restricting soil deformation. This structure can effectively transmit or dissipate stress, overcome the relative sliding between river silt particles and delay crack expansion and propagation. However, noticeable cracks developed in the immersed and F–T specimens. These microscopic characteristics can contribute to decreased mechanical properties for AFCS.

## Figures and Tables

**Table 1:** Physical properties of river silt used in this study.

Water Content /%	Density /(g/cm^3^)	Liquid Limit/%	Plastic Limit/%	Cohesion /kPa	Internal Friction /°	Specific Gravity	Organic Matter /%
70	1.70	60.5	47.9	35.8	8.77	2.26	0.62

**Table 2:** Physical properties used in this study.

Fiber	Color	Tensile Strength /MPa	Elongation /%	Diameter /μm	Density /(g/cm^3^)
Alginate fiber	White	320	10.97	16	0.91
Polypropylene fiber	White	460	10	150	1.31

**Table 3:** Chemical composition (wt.%) and physical properties of cement.

Chemical Composition	SiO_2_	CaO	Fe_2_O_3_	Al_2_O_3_	Na_2_O	K_2_O	MgO	SO_3_	Others	Loss on Ignition
	21.7	57.4	2.9	7.5	0.5	0.4	1.7	3.5	/	4.4
Physical properties	Initial setting time /min	Final setting time /min	Unconfined compressive strength /MPa		Specific surface area /(M^2^/kg)	Fineness /%	Particle size /%			
			3d	28d			<1 μm	1~3 μm	3~32 μm	>65 μm
	203	250	27.4	45	357	0.08	0	81.4	18.6	0

**Table 4:** Specific scheme.

Test Sample	Fibers	C/%	T/d	F/%	L/mm	Code Name
F–T cycle test	Alginate fibers; Polypropylene fibers	9	28	0, 0.6, 0.9	3, 6	C9F0L0-CS C9F0.6L3-AFCS C9F0.6L6-AFCS C9F0.9L6-AFCS C9F0.6L6-PFCS C9F0.9L6-PFCS
W–D cycle test	Alginate fibers	9	28	0.6, 0.9	6, 9	C9F0L0 C9F0.6L6 C9F0.6L9 C9F0.9L6 C9F0.9L9
Water immersion test	Alginate fibers	9	28	0.3, 0.6, 0.9	3, 6, 9	/
Seawater erosion test	Alginate fibers	9	7	0.6, 0.9	6	C9F0L0-Freshwater C9F0.6L6-Freshwater C9F0.9L6-Freshwater C9F0L0-Seawater C9F0.6L6-Seawater C9F0.9L6-Seawater
SEM test	Alginate fibers	9	28	0.3, 0.6, 0.9	3, 6, 9	/

Note: C = cement content (by weight of wet soil); T = curing time; F = fiber content (by weight of wet soil); L = fiber length (based on critical length).

**Table 5:** Main instruments and equipment used in experiments.

Test Name	Items Tested	Test Standard	Test Equipment	Manufacturer
F–T cycle test	UCS, mass loss	ASTM D560/D560M-16 [39], ASTM D2166-06 [40]	Naiheng Technology Durability Ace Series Concrete Rapid F–T Test System, Electronic universal testing machine	Beijing Naiheng Technology Co., Ltd. in Beijing, China, MTS Industrial Systems Co., Ltd. in Eden Prairie, MN, USA
W–D cycle test	UCS, mass loss, residual strength index	ASTM-D559-15 [41], ASTM D2166-06 [40]	Electronic universal testing machine	MTS Industrial Systems Co., Ltd. in Eden Prairie, MN, USA
Water immersion test	UCS, softening coefficient	Wang et al. [42], ASTM D2166-06 [40]	Electronic universal testing machine	MTS Industrial Systems Co., Ltd. in Eden Prairie, MN, USA
Seawater erosion test	UCS, degree of seawater erosion damage	Wu et al. [43], ASTM D2166-06 [40]	Electronic universal testing machine	MTS Industrial Systems Co., Ltd. in Eden Prairie, MN, USA
SEM imaging			NanoSEM450 Field Emission Scanning Electron Microscope	Oxford Instruments plc, in Oxford, UK

**Table 6:** Element (*W*_t_/%) of sample.

Type	C	O	Mg	Al	Si	S	K	Ca	Fe	Cr	Zr	Mo
AFCS	13.61	45.69	0.99	7.36	16.17	1.19	1.35	9.27	4.37	/	/	/
PFCS	24.33	25.70	0.57	4.24	8.90	/	/	7.13	6.28	0.30	20.02	2.54

**Table 7:** Parameters of residual strength index model.

F	L	a	b	c
0	0	−0.0477	0.2091	98.804
0.6	6	−0.0513	0.5094	100.39
0.6	9	−0.0587	0.7001	100.35
0.9	6	−0.0506	0.6111	101.20
0.9	9	−0.0509	0.7292	101.68

**Table 8:** Softening coefficient of AFCS.

Alginate Fiber Content (%)	Alginate Fiber Length (mm)	Standard Compressive Strength (kPa)	Immersed Compressive Strength (kPa)	Softening Coefficient (γ)
0.3	3	487.89	423.36	0.87
	6	493.01	437.99	0.89
	9	495.45	455.82	0.92
0.6	3	512.78	422.49	0.82
	6	524.28	474.12	0.90
	9	527.93	488.08	0.92
0.9	3	521.15	486.97	0.93
	6	529.7	499.37	0.94
	9	531.74	509.26	0.96

## Data Availability

Data are contained within the article.
